# Maternal risk factors for low birth weight for term births in a developed region in China: a hospital-based study of 55,633 pregnancies

**DOI:** 10.7555/JBR.27.20120046

**Published:** 2012-12-15

**Authors:** Yihua Bian, Zhan Zhang, Qiao Liu, Di Wu, Shoulin Wang

**Affiliations:** a State Key Laboratory of Reproductive Medicine, Institute of Toxicology, School of Public Health, Nanjing Medical University, Nanjing, Jiangsu 210029, China;; b Department of Reproductive Health Care, Wuxi Maternal and Child Health Hospital Affilaited to Nanjing Medical University, Wuxi, Jiangsu 214002, China.

**Keywords:** maternal factors, low birth weight (LBW), hypertensive disorders, multivariate regression analysis

## Abstract

Low birth weight (LBW) is an important risk factor for neonatal and infant mortality and morbidity in adults.. However, no large scale study on the prevalence of LBW and related maternal risk factors in China has been published. To explore the effects of maternal factors on LBW for term birth in China, we conducted a hospital-based retrospective study of 55, 633 Chinese pregnancy cases between 2001 and 2008. Maternal sociodemographic data, history of infertility and contraceptive use were obtained. Their medical status and diseases during pre-pregnancy were examined by physical examination at the first antenatal care visit. Maternal medical status before childbirth and pregnancy outcomes, including body weight, infant gender, multiple pregnancy and congenital anomalies, were recorded. Univariate and multivariate logistic regression, and linear regression were used to investigate the relationship between maternal factors and term LBW. The general incidence of term LBW was 1.70% in the developed area of China. After preliminary analysis using the univariate model, low primary education, anemia, hypertensive disorders, placental previa, oligohydramnios and premature rupture of membrane were predicted as independent factors of term LBW in the multivariate model. Furthermore, the decrease in annual frquencies of these risk factors were major causes of gradual decline in the incidence of LBW (from 2.43% in 2001 to 1.21% in 2008). The study demonstrated that among maternal factors, primary education, anemia and hypertensive disorders could contribute to LBW for term birth even in the most developed area of China.

## INTRODUCTION

Low birth weight (LBW), regardless of gestational age, is a multifaceted public health problem with significant individual and societal impact worldwide, especially in developing countries[1]. Globally, an estimated 20 million LBW infants are born each year, with over 18 million of these in developing countries. In 2004, the World Health Organization (WHO) reported that the average annual prevalence of LBW was approximately 6% in China. However, China contributes significantly to the overall number of LBW newborns by its large population. More than one million LBW infants are born each year in China. LBW infants are at a disproportionately higher risk of mortality, morbidity, poor growth, and impaired psychomotor and cognitive development[2],[3]. These LBW infants are also disadvantaged when they become adults as they are more susceptible to type 2 diabetes, hypertension, and coronary heart disease[4]. Infant mortality rate is six-fold higher in LBW infants and 100-fold higher in infants with very low birth weight (VLBW; i.e., < 1500 g) compared with normal birth weight infants[5]. The goal of “A World Fit for Children” sought to reduce the incidence of LBW of infants by at least one-third over 10 years, of which the deadline was 2010. The reduction in LBW of infants is also an important objective of Millennium Development Goal (MDG) 4, which seeks to reduce child mortality by two-thirds by 2015[6].

In China, as family planning policy was initiated in the 1970s, the health issue of children has gradually become a major concern, not only for individual families but also for the entire society. However, the currently high incidence of LBW is problematic. The high rates of mortality and morbidity arise from LBW, which impose an immense burden on health resources, education, social services and families[7]. Therefore, reduction of LBW rate is an urgent public health issue to avert immediate adverse outcomes in children and prevent global epidemics of diabetes, hypertension and cardiovascular diseases in adults, which are inversely related to birth weight.

LBW is not only related to basic maternal characteristics during pre-pregnancy, but also related to potential risk factors during pregnancy including maternal age, educational attainment, lifestyle, health status and diseases[2], of which maternal age, educational attainment and marital status are more closely associated with LBW. In addition, most current studies of LBW risk factors have focused on environmental, psychosocial, behavioral and medical factors. Behavioral risk factors that influence the incidence of LBW include smoking, drinking and illicit drug use[8]-[10]. Many medical factors and basic diseases are also reported to be related to LBW, including diabetes, preeclampsia, and oligohydramnios[11].

LBW can result from preterm birth (born before 37 weeks of gestation) or intrauterine growth restriction (IUGR). However, the definition of IUGR remains elusive as growth potential cannot be precisely quantified. Therefore, most cases of growth restriction are also considered small for their gestational age (SGA), but birth weight or estimated fetal weight can also be used as a surrogate[12]. This could be misleading because not all small babies are growth-restricted, and not all growth-restricted infants are small[13]. Presently, most studies have focused on infants born premature or SGA or included these risk groups in the sample without specific analyses of risk factors for LBW of single term birth.

To more precisely determine the maternal risk factors associated with LBW, we examined the relationship between birth weight and maternal characteristics (i.e., low primary education, diabetes mellitus, heart disease, viral hepatitis, thoracocyllosis, kidney disease during pre-pregnancy and disorders associated with pregnancy, such as anemia, hypertensive disorders, placental previa, oligohydramnios, uterine rupture and premature rupture of membrane (PROM)) in a cohort of 55,633 pregnant Chinese women who had a single full-term delivery between 2001 and 2008. The results may protect infants from these factors in maternal pre-pregnancy and through gestation.

## PATIENTS AND METHODS

### Study population

**Fig. 1:**
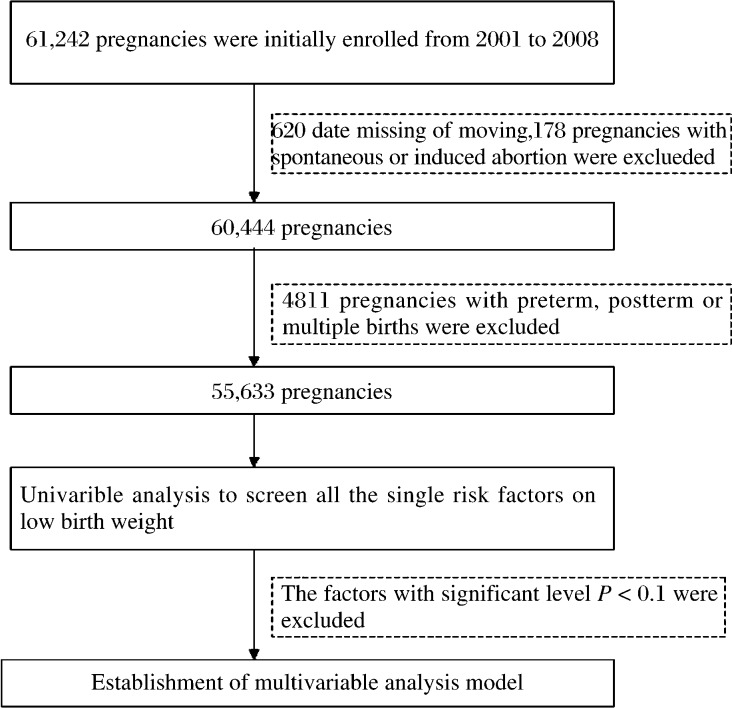
Study profile. After pregnancies with missing data, abortions, pre- and post-term births, and multiple births were excluded, a total of 55,633 pregnancies with single term births were analyzed in the present study.

This was a hospital-based retrospective study conducted between 2001 and 2008 at Wuxi Maternal and Child Health Hospital, affiliated with Nanjing Medical University, Wuxi, Jiangsu, China. All pregnant women whom came from Wuxi and its suburbs were asked to register when they first arrived at the hospital. Subsequently, each pregnant woman was asked to complete a structured questionnaire to provide individual information and a medical history. A series of regular medical examinations were conducted during pregnancy until delivery. A total of 61, 242 women participated in the investigation during the 8 years, of which 798 (1.3%) were excluded because of missing data, moving and spontaneous or induced abortion. Women with preterm delivery, post-term pregnancy (> 41 weeks) and multiple pregnancies (twins or more) were not included in the study as the data would interfere with LBW data. Thus, 55,633 pregnant women were enrolled in the study ***(Fig. 1)***.

### Information collection

The 8 year data for enrolled pregnant women were obtained from digital record database of the authors' hospital. In brief, each woman completed structured questionnaire to provide individual information, such as maternal sociodemographic data (i.e., age, education level, career opportunities, smoking, and alcohol consumption), history of infertility and contraceptive use. Their medical status and diseases (e.g., hypertension, diabetes mellitus, heart disease, viral hepatitis, thoracocyllosis, and kidney disease) during pre-pregnancy were examined by physical examination at the first antenatal care visit in the hospital. Maternal medical status before childbirth, such as hyperemesis gravidarum, fever, anemia, blood pressure, TORCH (toxoplasmosis, other viruses, rubella virus, cytomegalo virus and herpes virus), anomaly of the uterus, abnormalities of the amniotic fluid and uteroplacental disorders, were recorded and followed up until the babies were born and left the hospital. Pregnancy outcomes, including body weight, infant gender, multiple pregnancies and congenital anomalies, were also regularly recorded. All live newborns were evaluated by determining Apgar scores at 1 and 5 min. The data from the antenatal care unit and delivery room were collected. The present study was approved by the Institutional Review Board of Nanjing Medical University (Nanjing, Jiangsu 210029, China), and all procedures were in accordance with prevailing ethical principles.

### Definitions

In the present study, LBW was defined as delivery of single live newborn with birth weight < 2,500 g after a regular gestational period of 37–41 w. Hyperemesis gravidarum was defined as severe vomiting combined with positive urine ketone. Hypertensive disorders during pregnancy were divided into three specific subtypes: 1) gestational hypertension (blood pressure < 140/90 mmHg without proteinuria after 20 weeks of gestation); 2) mild preeclampsia (blood pressure ≥ 140/90 mmHg with proteinuria of 1^+^ on dipstick in two samples taken 6 h apart or > 300 mg in a 24 h urine collection); 3) severe preeclampsia (blood pressure ≥ 160/110 mmHg with proteinuria of 2^+^ on the dipstick in two samples taken 6 h apart or > 2 g total protein within a 24 hous urine collection). Anemia during pregnancy was defined as a hemoglobin concentration < 10 g/dL, which was tested after 8–10 weeks of gestation. Oligohydramnios was identified as an amniotic fluid volume < 300 mL. Polyhydramnios was defined as an amniotic fluid volume > 2,000 mL.

### Statistical analysis

CData were entered into computer and double-checked before analysis. All statistical analyses were performed using the SPSS 13.0 package (SPSS, Chicago, IL). LBW incidence associated with each factor was compared by χ^2^ test and Fisher's exact test by using a univariate analysis model. If the variables were statistically significant (*P* ≤ 0.10), they would be selected for further analysis of independent effects on LBW by using a multivariate analysis model with confounding variables controlled. Odds ratios (ORs) and 95% confidence intervals (CIs) were estimated by use of both univariate and multivariate analyses. Additionally, linear regression was applied to show variables including different years, LBW and related factors. regression coefficients and significance levels were also calculated.

## RESULTS

As shown in ***Fig. 1***, a total of 55,633 pregnant women, aged 20 to 44 years, were enrolled after preterm, post-term, and multiple birth pregnancies were excluded. The incidence of LBW was 1.7%. The mean age of women was 26±3 years, and the mean infant birth weight was 3374±415 g with the range of 1,000 to 5,900 g. Among neonates, 98.6% were evaluated as healthy, determined by Apgar scores with 1 or 5.

***Table 1*** summarizes the distribution of incidence of LBW associated with basic maternal characteristics and health status during pre-pregnancy. The incidences of LBW with regard to low primary education (2.20%, OR = 1.44), diabetes mellitus (14.29%, OR = 9.66), heart disease (4.08%, OR = 2.47), viral hepatitis (5.66%, OR = 3.48), thoracocyllosis (6.56%, OR = 4.07), and kidney disease (5.26%, OR = 3.22) were significantly higher than their individual references (*P* < 0.05 or *P* < 0.01). The results indicated that the above factors were related to higher risks for LBW. Among these risk factors, diabetes mellitus was associated with the highest incidence of LBW, which was approximately 8.4-fold higher than that of women without diabetes mellitus (14.29% vs. 1.70%).

**Table 1:** Univariate analysis of basic maternal characteristics during pre-pregnancy and the incidence of LBW

	N	LBW		χ^2^	OR	*P* value
		N	%			
Age (years)						
≥35	648	11	1.70	0.00	1.00	1.00
< 35	54985	934	1.70			
Education						
primary (≤ 9 years)	13793	303	2.20	27.25	1.44	< 0.01
high (> 9 years)	41840	642	1.53			
Maternal smoking						
yes	38	0	0.00	0.66	NA	0.42^a^
no	55595	945	1.70			
Passive smoking						
yes	1672	32	1.91	0.48	1.13	0.51
no	53961	913	1.69			
Alcohol use						
yes	73	0	0.00	1.26	NA	0.26^a^
no	55560	945	1.70			
History of infertility						
yes	468	6	1.28	0.49	0.75	0.59
no	55165	939	1.70			
Contraceptives use						
yes	261	5	1.92	0.07	1.13	0.79^a^
no	55372	940	1.70			
Hypertension						
yes	14	1	7.14	2.49	4.46	0.12^a^
no	55619	944	1.70			
Diabetes mellitus						
yes	14	2	14.29	13.29	9.66	< 0.01^a^
no	55619	943	1.70			
Heart disease						
yes	147	6	4.08	5.01	2.47	0.03^a^
no	55486	939	1.69			
Viral hepatitis						
yes	53	3	5.66	4.99	3.48	0.03^a^
no	55580	942	1.69			
Thoracocyllosis						
yes	61	4	6.56	8.63	4.07	< 0.01^a^
no	55572	941	1.69			
Kidney disease						
yes	57	3	5.26	4.34	3.22	0.04^a^
no	55576	942	1.69			

a: calculated with Fisher's Exact Test. LBW: low birth weight; NA: not applicablett.

The influence of disorders during pregnancy on LBW was also investigated. As shown in ***Table 2***, anemia (2.48%, OR = 1.51), hypertensive disorders (5.03–15.04%, OR = 3.35–10.03), placental previa (5.03%, OR = 3.08), oligohydramnios (3.01%, OR = 1.83), and fetal distress (2.60%, OR = 1.60) were associated with a much higher risk for LBW (*P* < 0.01). Uterine rupture (4.76%, OR = 2.90) and PROM (2.14%, OR = 1.29) also had a certain risk for LBW (*P* < 0.05). Among these risk factors, severe preeclampsia in hypertensive disorders was associated with the highest incidence of LBW, which was approximately 10-fold higher than that in normotensive women (15.04% vs. 1.50%).

**Table 2:** Univariate analysis of maternal characteristics during pregnancy and the incidence of LBW

	N	LBW		χ^2^	OR	*P* value
		N	%			
Hyperemesis gravidarum						
yes	661	11	1.66	0.01	0.98	1.00
no	54972	934	1.70			
Fever						
yes	118	1	0.85	0.51	0.49	0.47^a^
no	55515	944	1.70			
Exposure to risk factors						
yes	67	1	1.49	0.02	0.88	0.90^a^
no	55622	944	1.70			
Anemia						
yes	2777	69	2.48	10.82	1.51	<0.01
no	52856	876	1.66			
Hypertensive disorders during pregnancy						
severe preeclampsia	113	17	15.04	217.69	10.03	<0.01
mild preeclampsia	359	26	7.24		4.83	
gestational hypertension	735	37	5.03		3.35	
normotensive	54426	817	1.50			
TORCH						
yes	317	8	2.52	1.30	1.50	0.27
no	55316	937	1.69			
Myoma of the uterus						
yes	37	2	5.41	3.05	3.18	0.08^a^
no	55596	943	1.70			
Rupture of the uterus						
yes	84	4	4.76	4.73	2.90	0.03
no	55549	941	1.69			
Premature rupture of membrane						
yes	3361	72	2.14	4.22	1.29	0.04
no	52272	873	1.67			
Placental abruption						
yes	94	3	3.19	1.26	1.91	0.26^a^
no	55539	942	1.70			
Placental previa						
yes	179	9	5.03	11.92	3.08	<0.01^a^
no	55454	936	1.69			
Polyhydramnios						
yes	190	4	2.11	0.19	1.25	0.66^a^
no	55443	941	1.70			
Oligohydramnios						
yes	1163	35	3.01	12.22	1.83	<0.01
no	54470	910	1.67			
Cord entanglement						
yes	2757	47	1.70	0.00	1.00	0.95
no	52876	898	1.70			

a: calculated with Fisher's Exact Test. LBW: low birth weight; TORCH: toxoplasmosis, other viruses, rubella virus, cytomegalo virus, herpes virus.

Fever: ≤38.5°C at least 24 hours.

**Fig. 2:**
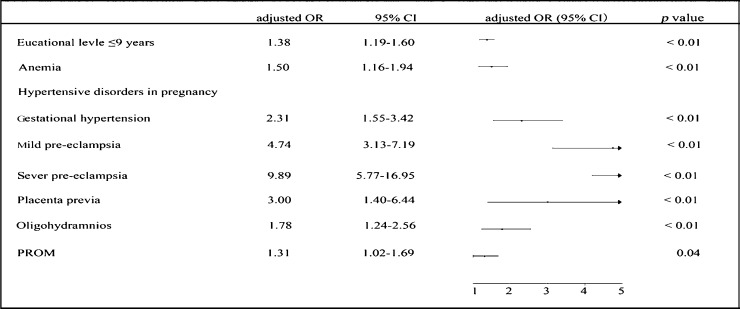
Multivariate logistic regression analysis of candidate factors related to term LBW. The OR was adjusted by neonate gender and maternal age. LBW: low birth weight; PROM: premature rapture of membrane.

Based on a significance level of *P* ≤ 0.10, candidate factors or exposures during pre-pregnancy and perinatal stage were selected to analyze their individual influence on the incidence of LBW by using a multivariate regression model. ***Fig. 2*** summarizes independent factors that increased the risk for LBW, including primary school education (≤ 9 years), anemia, hypertensive disorders, placental previa, oligohydramnios and PROM. All factors had significant adverse effects on the incidence of LBW, even when adjusted for infant gender. Among these factors, low education level increased the incidence of LBW by 38% (*P* < 0.01), indicating that high education level may decrease the incidence of LBW, especially in China. Other factors including anemia, placental previa, oligohydramnios and PROM also increased the incidence of LBW by 31%–200% (*P* < 0.05).

**Fig. 3:**
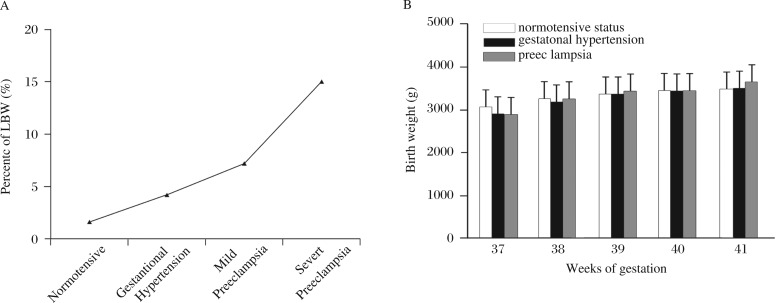
Effects of hypertensive disorders on term low birth weight (LBW). A: Relationship between the incidence of LBW and hypertensive disorders at different gestational ages. Hypertensive disorders in gestation are classified as normotensive, gestational hypertension, mild preeclampsia and sever preeclampsia. B: Birth weight associated with normotensive status and hypertensive disorders (gestational hypertension and preeclampsia) at term gestational ages (37–41 weeks).

Hypertensive disorders during pregnancy might be associated with a much higher risk for LBW (OR = 4.83, *P* < 0.01) ***(Table 1)***. Compared with normotensive status, the incidence of LBW associated with gestational hypertension, mild preeclampsia and severe preeclampsia were 5.03% (OR = 3.35), 7.24% (OR = 4.83), and 15.04% (OR = 10.03), respectively (*P* < 0.01), which showed a positive relationship between symptoms and risk ***(Table 2, Fig 3A)***. This association was also found in multivariate regression analysis. Regardless of whether the data were adjusted by infant gender, hypertensive disorders increased the risk for LBW (OR = 2.31–9.89, *P* < 0.01; ***Fig. 2***). Analysis of the effects of hypertensive disorders on term LBW during different gestational weeks showed that only during 37–38 weeks, birth weight in the normotensive group was significantly higher than that in the gestational hypertension or preeclampsia group ***(Fig. 3B)***. These results indicated that hypertensive disorders, including gestational hypertension and preeclampsia, might be one of the most important risk factors for term LBW.

**Fig. 4:**
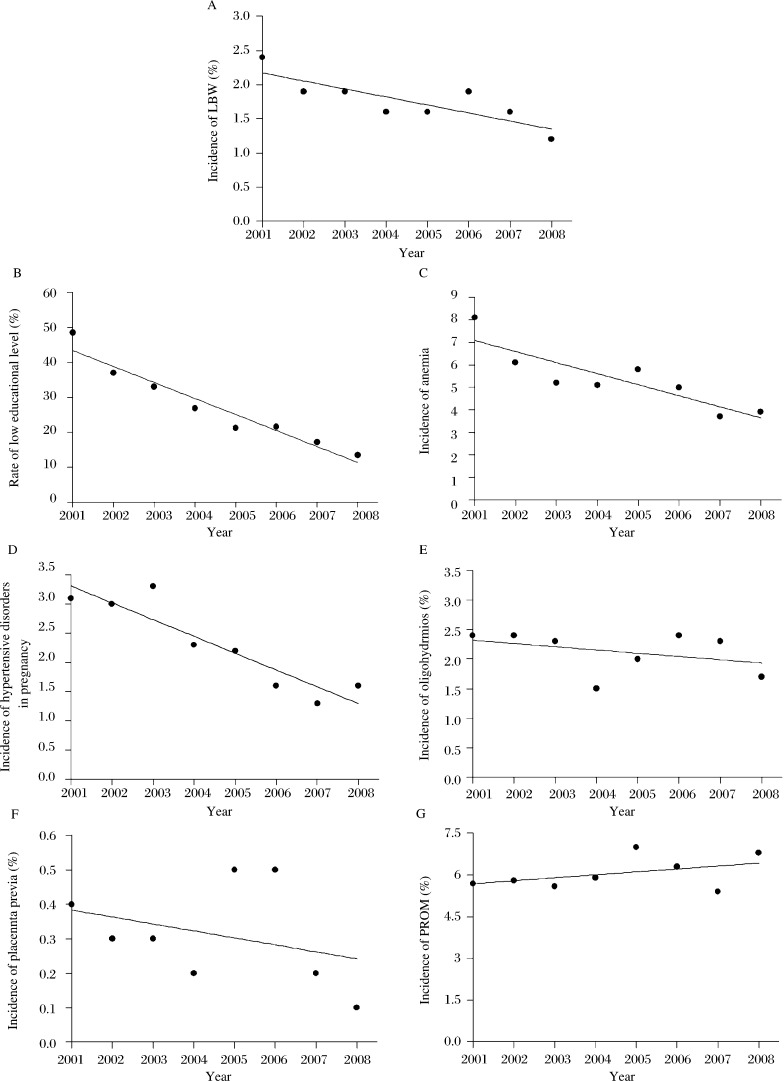
Linear regression between years and the incidence of low birth weight and its candidate risk factors. A: Linearity of years with LBW incidence. Linearity of years with candidate risk factors, including low education level (B), anemia (C), hypertensive disorders (D), oligohydramnios (E), placental previa (F) and premature rapture of membrane (PROM) (G).

Additionally, the incidence of LBW showed a downward trend from 2.43% in 2001 to 1.21% in 2008. A significant relationship was found between years and the incidence of LBW (R^2^ = 0.68, P = 0.01; ***Fig. 4A***). Interestingly, the incidences of factors were also dependently decreased by year. Among these, the rates of low primary education (R^2^ = 0.94, *P* < 0.01; ***Fig. 4B1***), anemia (R^2^ = 0.76, *P* < 0.01; ***Fig. 4B2***) and hypertensive disorders during pregnancy (R^2^ = 0.85, *P* < 0.01; ***Fig. 4B3***) showed a significant decline by year. However the incidence of placental previa ***(Fig. 4B4)***, oligohydramnios ***(Fig. 4B5)*** and PROM (***Fig. 4B6***) did not show the same trend. These results suggested that decreased incidence of LBW might be associated with a decline in primary education, anemia, hypertensive disorders and fetal distress from 2001 to 2008.

## DISCUSSION

LBW is regarded as an adverse pregnancy outcome associated with many risk factors during pre-pregnancy and gestation. Many previous studies have demonstrated that preterm and multiple pregnancies were associated with LBW[14]-[16]. With the exception of congenital malformations, preterm births result in 75% of neonatal deaths and more than half of long-term morbidity[17]. Use of assisted reproductive technology leads to the increase in the rate of multiple pregnancies[18]. Multiple births represent 3% of all deliveries but account for 15% of preterm births, 20% of LBW and 19%–24% of VLBW infants in the United States[19]. The above factors are well known to be associated with LBW. Therefore, when we focused on LBW among single term births in the present study, preterm, post-term and multiple births were excluded.

Basic characteristics, diseases during pre-pregnancy, disorders during gestation were analyzed by the present study to determine their association with the incidence of LBW. Although more than 30 variables were analyzed, only 12 showed a significant influence on LBW in the univariate regression model. A low education level (≤ 9 years), anemia, hypertensive disorders during pregnancy, placental previa, oligohydramnios and PROM were found to contribute to the incidence of LBW in the multivariate regression model, adjusted for maternal age and infant gender.

Anemia is a common nutritional deficiency disorder and is very usual in pregnant women worldwide[20]. Prevalence of anemia in pregnant women in developing countries is higher than in developed countries. A previous study reported that maternal anemia was associated with fetal anemia and stillbirth and further affected embryo development, leading to LBW[21]. Maternal anemia is not only responsible for maternal mortality but also associated with preterm birth and the incidence of LBW[22]. In the present study, the incidence of LBW in women suffered from anemia during pregnancy was about 1.5-fold of that in normal pregnancies, suggesting that improving nutrition status during pregnancy is very important for controlling LBW.

Hydramnios and oligohydramnios are defined as excessive or deficient amniotic fluid. They are associated with an increase in perinatal mortality and have been correlated with smaller gestational age and lower birth weight[23]. Consistent with a previous report[24], oligohydramnios was a risk factor for LBW in the present study. Oligohydramnios might cause fetal anoxia and affect growth to induce LBW. Placenta previa and placental abruption are both important placental pathologies. They are believed to have a similar etiology of LBW[25] and both lead to perinatal mortality and morbidity[26]. Placenta previa is a known risk factor for antepartum hemorrhage and preterm delivery. Pregnancies complicated by placenta previa are more likely to deliver infants with LBW[27]. Several studies have reported that placental abruption is associated with LBW, preterm birth and IUGR[28]. In the present study, placenta previa, but not placental abruption, increased the risk for LBW.

Approximately 6%–8% of all pregnancies are complicated by hypertensive disorders[29]. Gestational hypertension, which is classified as non-proteinuric or proteinuric (mild preeclampsia and severe preeclampsia), has a major influence on maternal and neonatal morbidity and mortality[30]. Previous studies showed that preeclampsia was associated with LBW and preterm birth. However, the rate of fetal growth restriction was 10%–25% in severe preeclampsia. Preeclampsia and gestational hypertension influenced birth weight by gestational age. These results indicated that hypertensive disorders might play a critical role in the incidence of LBW. Although the incidence of LBW was significantly restricted by preterm birth, it still showed a positive relationship with hypertensive disorders in women with term deliveries. Unexpectedly, diabetes was not in the multivariate model although OR was the highest (OR = 9.66, *P* < 0.01) ***(Table 2)***. Except for the number of women with diabetes is very low (14 vs. 55619), two women with LBW were included in women with hypertensive disorder, which suggested that diabetes might be an adjunctive factor of hypertensive disorder rather than an independent factor of LBW.

The incidence of term singleton infants with LBW in Wuxi was 1.70%. When preterm birth data were included in analysis, the incidence was 3.30%, which seemed lower than the average level in China and worldwide. Nonetheless, the incidence of LBW gradually declined from 2.43% (5.50% for total LBW) in 2001 to 1.21% (2.50% for total LBW) in 2008, which showed a significant linear regression. Interestingly, the incidences of the selected factors, such as low educational level, anemia and hypertensive disorders have decreased annually, which seem to be a good explanation for the decline of the incidence of LBW in this area. These changes further proved that the incidence of LBW was strongly associated with the given risk factors.

To our knowledge, our cohort size was the largest of the studies that have analyzed the effects of maternal factors on the incidence of LBW for term birth in China. LBW is a multifactorial outcome and remains a public health problem. It might be a potential risk factor for poor growth and development in children and some diseases in adults. Therefore, a follow-up epidemiologic investigation has been planned, which will help protect healthy children from the influence of LBW, as health of children is the most concerned problem for families since Chinese family planning policy was initiated, especially in the most developed area of China.
